# Factors influencing frequency and severity of human-American black bear conflicts in New York, USA

**DOI:** 10.1371/journal.pone.0282322

**Published:** 2023-02-24

**Authors:** Jamshid Parchizadeh, Kenneth F. Kellner, Jeremy E. Hurst, David W. Kramer, Jerrold L. Belant

**Affiliations:** 1 Department of Fisheries and Wildlife, Michigan State University, East Lansing, MI, United States of America; 2 New York State Department of Environmental Conservation, Albany, NY, United States of America; Cheetah Conservation Fund, Namibia University of Science and Technology, NAMIBIA

## Abstract

Free-ranging large carnivores are involved in human-wildlife conflicts which can result in economic costs. Understanding factors that lead to human-wildlife conflicts is important to mitigate these negative effects and facilitate human-carnivore coexistence. We used a human-American black bear (*Ursus americanus*) conflict database maintained by the New York State Department of Environmental Conservation to determine whether drought, conflicts within the Adirondack and Catskill Parks as compared to outside of these parks, mild severity (Class 3) conflicts early in the year (April–June), and bear harvest in the previous year (as an index of bear abundance), were associated with greater frequency of high or moderate severity (Class 1–2) conflicts later in the year (July–September) across New York, USA. During 2006–2019, we obtained 3,782 mild severity conflict records early in the year, and 1,042 high or moderate severity records later in the year. We found that a one standard deviation increase in the cumulative precipitation difference from mean early in the year (about 7.59 cm) coincided with a 20% decrease in conflicts, and that Wildlife Management Units (WMUs) within the parks were predicted to have 5.61 times as many high or moderate severity conflicts as WMUs outside the parks. We also found that a one standard deviation increase in the frequency of mild severity conflicts (equivalent to 5.68 conflicts) early in the year coincided with an increase in the frequency of high or moderate severity conflicts in a WMU later in the year by 49%, while a one standard deviation increase in the bear abundance index in the previous year (0.14 bears/10 km^2^) coincided with a 23% increase in high or moderate severity conflicts. To reduce the frequency and severity of conflicts to facilitate human-black bear coexistence, we recommend the following measures to be taken in place consistently and build over time in local communities: (i) further reducing black bear access to anthropogenic foods and other attractants, (ii) non-lethal measures including bear-resistant waste management, (iii) electric fencing, and (iv) modifying placement or configuration of field crops.

## Introduction

Coexistence between people and free-ranging large carnivores poses one of the greatest conservation challenges of our time [1]. Where large carnivores co-occur with people, human-carnivore conflicts can be common, potentially endangering human safety and resulting inconsiderable economic costs [2–4]. Real or perceived human-large carnivore conflicts often end with carnivore mortalities and can reinforce negative attitudes toward these animals with long-term conservation consequences [5–7]. In extreme circumstances, these conflicts can lead to attacks on people resulting in human injury or death [8–10]. It is therefore important to understand mechanisms leading to human-carnivore conflicts such that mitigation strategies can be developed to facilitate coexistence.

In North America, American black bears (*Ursus americanus*) are the bear species most commonly involved in conflicts with humans [11–13]. Human-bear conflicts include crop damage, livestock depredation, destruction of property, and perceived and real threats to personal safety, though most conflicts are non-life threatening to humans [11, 14, 15]. In recent decades, populations of black bears and humans have increased throughout portions of the United States including some of the eastern states and consequently, increased human-black bear conflicts have been reported [16–18].

Drought can limit vegetation production [19–21], and therefore natural foods for wildlife including black bears [22, 23]. Scarcity of natural foods with high caloric energy (e.g., nuts and berries) may cause bears to leave their primary habitat and use anthropogenic foods in human-dominated landscapes, which in turn can result in increased conflicts with humans [12, 13, 24]. Proximity of wildlands and residential food sources for bears present a risk of conflict with people [25–27]. Consequently, human food conditioning by black bears, and unsecured foods and edible garbage are root causes of human-bear conflicts, and are also the primary factors that can be managed to facilitate coexistence [28–31].

Our objective was to examine how spatial and temporal variation in environmental factors are associated to incidence of high or moderate severity (Class 1–2) human-black bear conflicts later in the year (July–September) across New York, USA. First, we predicted that drought would coincide with an increased frequency of high or moderate severity conflicts. Second, we predicted that conflicts would be greater within two large New York parks (i.e., Adirondack and Catskill), which contained a mixture of public and private lands. Third, we predicted that there would be a positive association between mild severity (Class 3) conflicts early in the year (April–June), and the frequency of high or moderate severity conflicts later in the year. Finally, we predicted that the number of bears harvested in the previous year (as an index of bear abundance) would be positively associated with high or moderate severity conflicts in the current year.

## Materials and methods

### Study area

**Fig 1:**
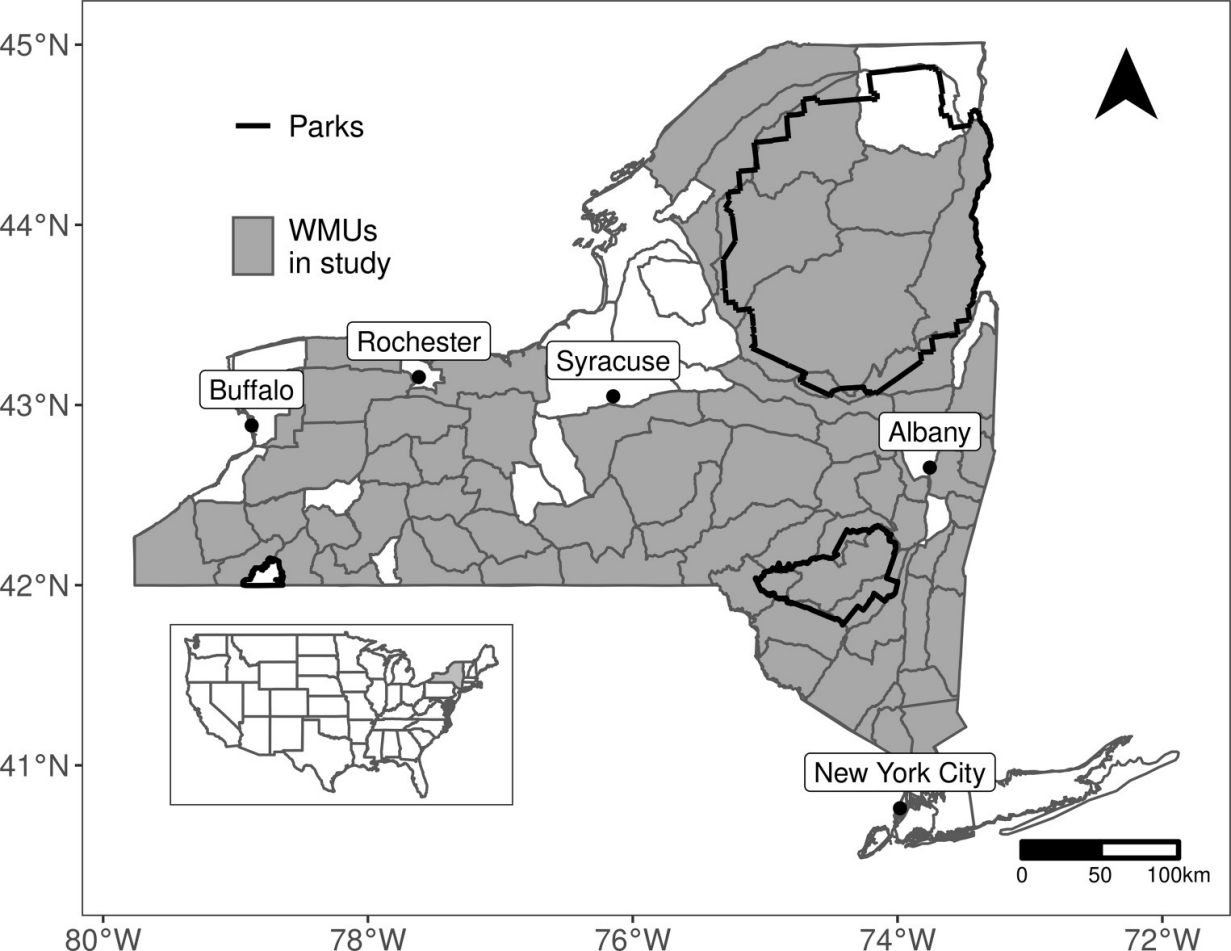
Locations of 68 Wildlife Management Units from which we obtained human-black bear conflict data, New York, USA. Major park boundaries are in black.

New York state is in the northeastern United States (40°29’40"–45°0’42"N, 71°47’25"–79°45’54"W) and comprises 141,299 km^2^ (Fig 1). It is the fourth most populous (20,201,249 people) state in the United States [32]. New York is characterized by a humid continental climate with average temperatures ranging from -9–1°C in January and 19–25°C in July [33, 34]. Mean annual precipitation is 1,016 mm, with relatively drier conditions in western regions and more moist conditions in northern regions (particularly the Adirondack Mountains [34]). Our study area included two major parks: the Adirondack Park which encompasses 19,700 km^2^ in northern New York, and the Catskill Park which encompasses 2,500 km^2^ in southeastern New York. An important and distinguishing feature of these two parks is that both contain a mixture of public and private lands.

### Conflict data

We used a human-black bear conflict database maintained by the New York State Department of Environmental Conservation (NYSDEC) during 2006–2019. Data on human-bear conflicts is recorded by the NYSDEC by compiling information from their responses to complaints submitted by the public. For each recorded conflict, the NYSDEC noted the type of complaint (e.g., bird feeder interaction, vehicle break-in, structural damage, etc.), location (i.e., Wildlife Management Unit (WMU), which is the spatial extent at which black bears are managed in New York and at which human-bear conflicts are often considered), and the severity of the conflict (denoted by classes 1 to 3 [13, 35]). Based on the NYSDEC’s severity classification system, Class 1 conflicts are highly severe (e.g., bear entered occupied or unoccupied homes, attacked pets or livestock, or displayed aggressive behavior toward people), Class 2 conflicts are moderately severe (e.g., human habituation and/or food conditioning), and Class 3 conflicts are mildly severe (e.g., bear feeding at a bird feeder, or raiding a dumpster or garbage can). For each year and WMU, we calculated the total frequency of mild severity (Class 3) conflicts that occurred early in the year (April–June), and the total frequency of high or moderate severity (Class 1–2) conflicts that occurred later in the year (July–September). We excluded from analysis WMUs that averaged less than one reported conflict per year during April–June. Our final dataset included 68 WMUs, which averaged 1,495 km^2^ in size (standard deviation = 1,227 km^2^; Fig 1).

### Covariates

For each WMU and year, we collected three additional covariate values: (i) a metric representing degree of drought severity early in the year, (ii) abundance index as a metric of relative bear population size, and (iii) a binary variable indicating if a given WMU was within a park. We calculated the drought metric as the cumulative difference in precipitation from the 30-year mean we derived using monthly total precipitation data for April–June 2006–2019 from 673 weather stations throughout New York [36]. We obtained 30-year (1981–2010) precipitation means for the same stations and calculated the difference from the mean for each monthly precipitation total for each station and year. We used kriging interpolation [37] with station-level data in each month and year to predict precipitation difference from means in a grid of 1-ha cells across New York, then averaged the 1-ha cells in each WMU to obtain an average precipitation difference from mean value for each WMU, month, and year. We used the cumulative difference in precipitation from the mean in June as the precipitation covariate for the April–June period in each WMU.

A statewide bear hunt occurs during fall within WMUs in New York including the Adirondacks and Catskill Parks, and there is no statewide quota. We used NYSDEC bear harvest records from the previous fall hunting season (11 September–21 December) as an index of relative bear abundance. During our study, hunting regulations were consistent and all licensed hunters (average 568,300 hunters during 2006–2019) were eligible to harvest a black bear, and most bears are taken opportunistically by deer hunters [35]. With such extensive potential hunting effort and an average annual harvest of only 1,344 bears during 2006–2019, bear harvest density and distribution are used by bear managers as an index of population trends [35].

Successful bear hunters are required by law to report their harvest. Furthermore, NYSDEC contacts individuals who report a bear harvest and provides them with a patch should they provide NYSDEC with a premolar tooth for age determination. Therefore, it is likely that the reporting rate is greater than that of deer (about 50%) in New York given the incentive to report.

We aggregated harvest records by WMU and year to obtain yearly harvest densities. For the park covariate, we identified WMUs for which all or most of the area occurred within Adirondack Park or Catskill Park.

### Analysis

We modeled total high or moderate severity conflicts in a given WMU and year as a function of combinations of our covariates. As the response variable was a count and overdispersed, we used negative binomial regression for all models. We defined five candidate regression models: (1) a null model with no covariates (NULL), (2) total mild severity conflicts early in the year and bear abundance index (PRIOR+POP), (3) mild severity conflicts, abundance, and precipitation difference from means (PRIOR+POP+PRECIP), (4) mild severity conflicts, abundance, and parks (PRIOR+POP+PARK), and (5) a global model (PRIOR+POP+PRECIP+PARK). We standardized continuous covariates to have a mean of 0 and a standard deviation of 1 before analysis. We included random intercepts by WMU in all models to help control for additional variation in conflicts by WMU (e.g., WMU area). We ranked models by AICc and retained for further consideration all models within ΔAICc = 2 of the top-ranked model. For each model, we considered a covariate to have had a significant effect if the corresponding Wald test had p< 0.05. We fit all models using the glmmTMB package [38] in R 4.1.1 [39].

## Results

**Fig 2:**
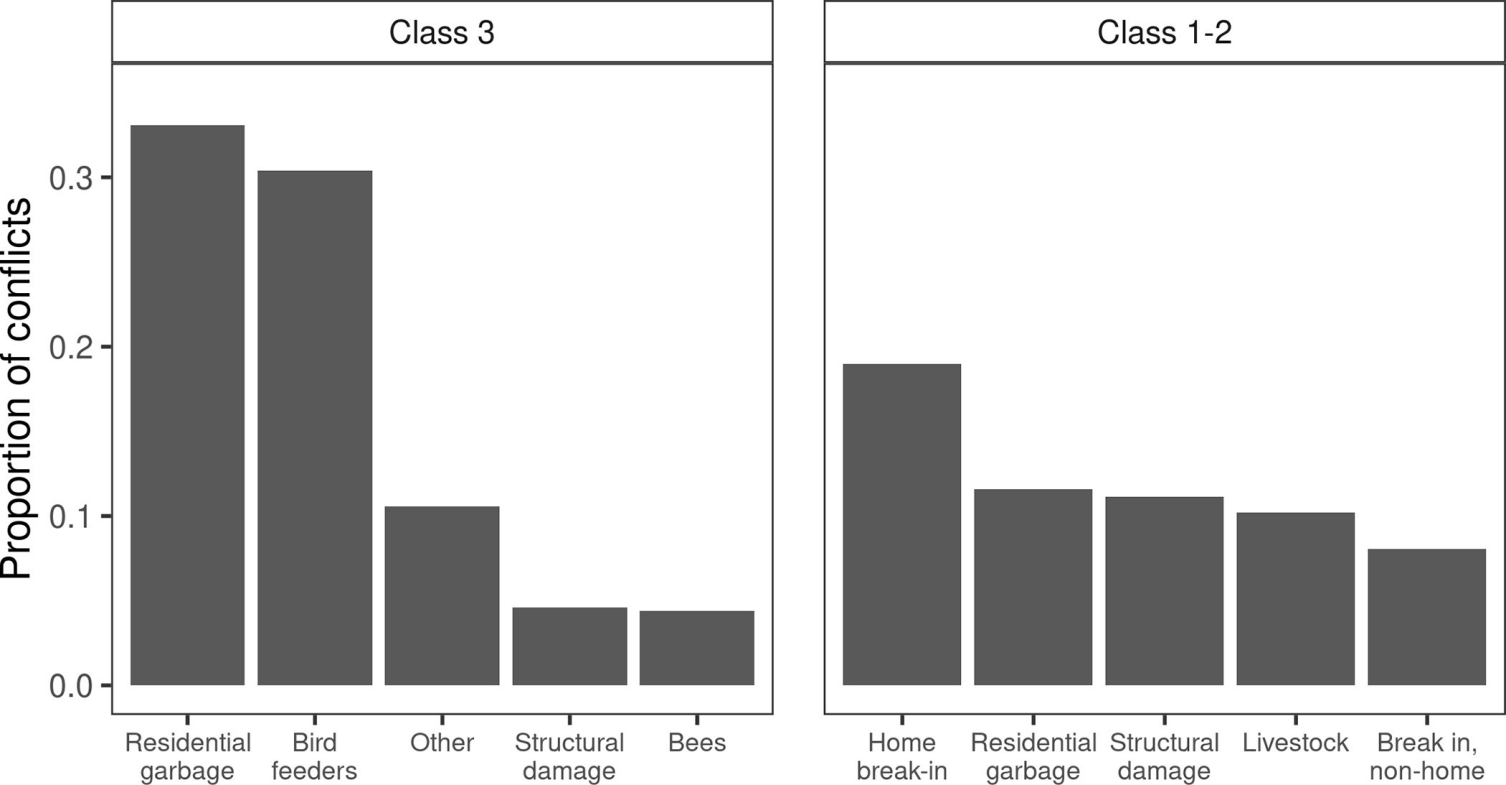
Proportions of the five most common types of reported human-black bear conflicts across 68 Wildlife Management Units, New York, USA, 2006–2019.

During 2006–2019 there were 3,782 mild severity conflicts reported early in the year, and 1,042 high or moderate severity conflicts reported later in the year. The most common types of mild severity conflicts involved residential garbage and bird feeders, whereas the most common types of high or moderate severity conflicts involved home break-ins or bears that were attracted to residential garbage but displayed undesirable behaviors in the presence of humans (Fig 2). The average number of mild severity conflicts early in the year for a given WMU and year was 4.5 (range 0–44), while the average number of high or moderate severity conflicts later in the year was 1.2 (range 0–62).

**Fig 3:**
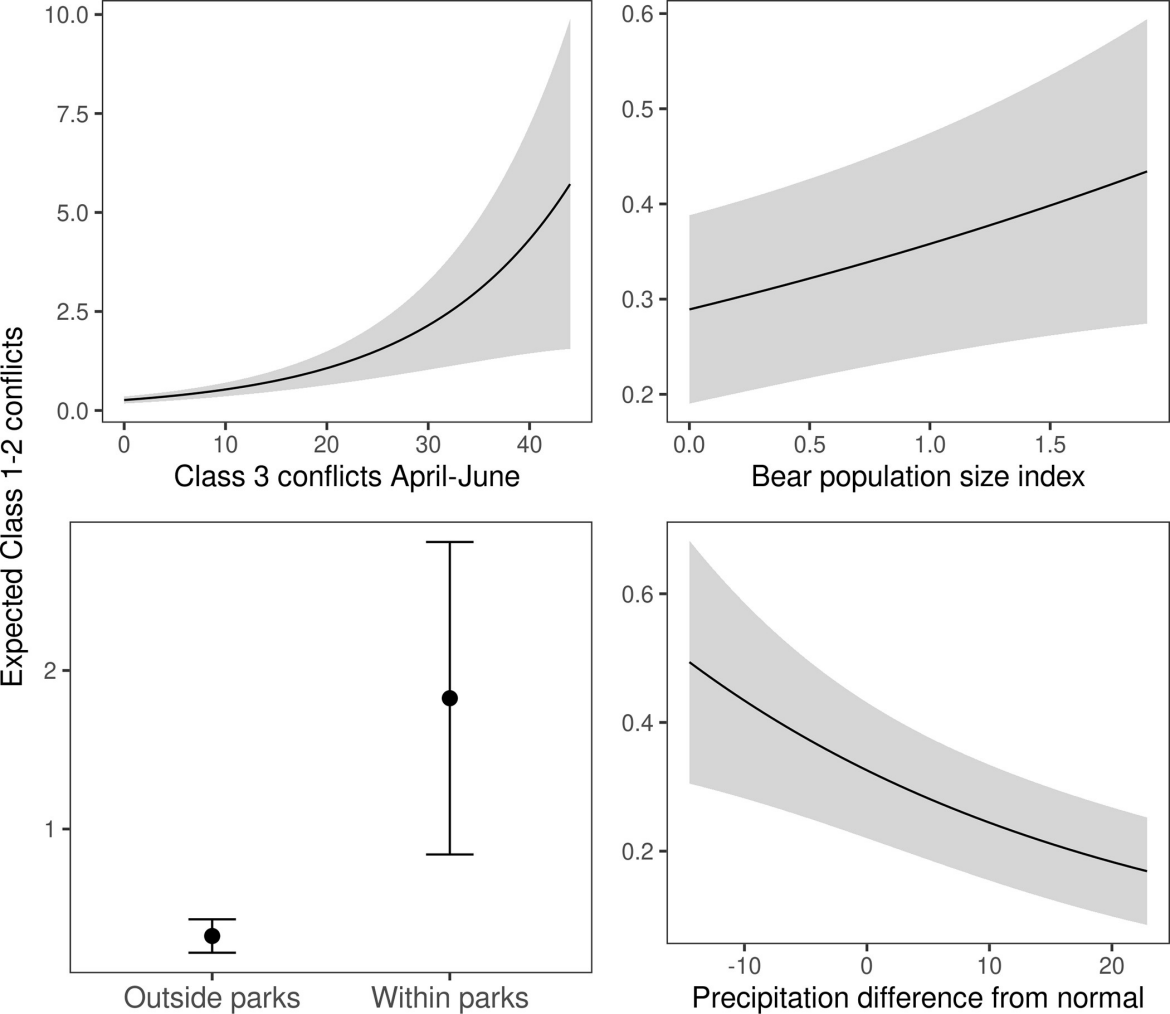
Effects of covariates included in the top model on high or moderate severity (Class 1–2) human-black bear conflicts, New York, USA, July–September 2006–2019. Shaded area and error bars represent 95% confidence intervals. For each panel, non-focal covariates were held at median values (mild severity conflicts, abundance index, precipitation) or 0 (protected status).

The global model (PRIOR+POP+PRECIP+PARK) had the most support among candidate models (Table 1). We found a significant effect of the frequency of mild severity conflicts, bear abundance index, and conflicts within the Adirondack and Catskill Parks on the frequency of high or moderate severity conflicts (Table 2, Fig 3). A one standard deviation increase in the frequency of mild severity conflicts (equivalent to 5.68 conflicts) early in the year coincided with an increase in the frequency of high or moderate severity conflicts in a WMU later in the year by 49%, while a one standard deviation increase in the bear abundance index in the previous year (0.14 bears/10 km^2^) coincided with a 23% increase in high or moderate severity conflicts. A one standard deviation increase in the cumulative precipitation difference from mean early in the year (about 7.59 cm) coincided with a 20% decrease in high or moderate severity conflicts. Wildlife Management Units within the parks were predicted to have 5.61 times as many high or moderate severity conflicts as WMUs outside of the parks.

**Table 1:** Results of model selection for negative binomial regression models of high or moderate severity (Class 1–2) bear conflicts, New York, USA, July–September 2006–2019. We considered five candidate models: (a) no covariates (NULL), (b) mild severity (Class 3) conflicts early in the year (April–June) and bear abundance index (PRIOR+POP), (c) mild severity conflicts, abundance, and precipitation difference from means (PRIOR+POP+PRECIP), (d) mild severity conflicts, abundance, and parks (PRIOR+POP+PARK), and (e) a global model (PRIOR+POP+PRECIP+PARK). Models were ranked using Akaike Information Criterion for small samples (AICc).

Model	Parameters	AICc	ΔAICc	Weight
PRIOR+POP+PRECIP+PARK	7	1706.38	0.00	1.00
PRIOR+POP+PARK	6	1718.05	11.67	0.00
PRIOR+POP+PRECIP	6	1730.23	23.84	0.00
PRIOR	5	1740.71	34.33	0.00
NULL	3	1792.05	85.67	0.00

**Table 2:** Results from a top-ranked negative binomial regression model of high or moderate severity (Class 1–2) bear conflicts, New York, USA, July–September 2006–2019. Covariates included mild severity (Class 3) conflicts early in the year (April–June; PRIOR), bear abundance index as a metric of relative bear population size (POP), cumulative precipitation difference from mean early in the year (PRECIP), and whether the Wildlife Management Unit (WMU) was within a park (PARK). Continuous covariates were standardized before analysis.

Parameter	Estimate	95% CI	*P*
Intercept	-0.97	-1.29 –-0.64	< 0.01
PRIOR	0.40	0.30–0.50	< 0.01
POP	0.20	0.06–0.35	< 0.01
PRECIP	-0.22	-0.34 –-0.10	< 0.01
PARK	1.72	1.13–2.32	< 0.01
Random WMU variance	0.53		
Overdispersion parameter	3.35		

## Discussion

Our predictions of patterns influencing the frequency and severity of human-American black bear conflicts in New York, USA, were largely supported. We found that drought was negatively corresponded with the frequency of high or moderate severity conflicts later in the year, and that conflicts were greater within the Adirondack and Catskill Parks. Our findings also suggested a positive association between mild severity conflicts early in the year and the frequency of high or moderate severity conflicts later in the year. Furthermore, greater bear abundance, as indicated by the size of the bear harvest the preceding fall, was positively associated with the frequency of high or moderate severity conflicts. The link between bear abundance and conflict may be present in New York because of inconsistent application of measures to facilitate human-black bear coexistence, and as a result to mitigate conflicts. Despite widespread educational campaigns of NYSDEC and laws prohibiting intentional feeding of bears, the New York public has not uniformly adopted wise practices to reduce attractants for bears around their homes and businesses. Consequently, in our study area, when more bears are present to capitalize on the food resources made available by the public, high or moderate severity conflicts increased.

Precipitation had a negative effect on the frequency of high or moderate severity conflicts. Reduced precipitation can lead to drought and reduced abundance and availability of natural foods for black bears [23, 40, 41], including mast crops [24]. Reduced abundance of natural foods can cause bears to seek human-derived foods, which could lead to increased conflicts [42, 43]. Conflicts between bears and humans increased throughout New York, USA, during summer droughts when berries were less abundant [35]. Similar switching between natural and anthropogenic foods due to reduced natural food availability during droughts has been reported for other taxa and bear species in other systems. Culpeo (*Lycalopex culpaeus*) and South American gray fox (*L*. *griseus*) shifted from wild to domestic prey (i.e., sheep) during droughts, when small mammal abundance was low [44]. Sun bears (*Helarctos malayanus*) altered their ranges across seasons and years based on natural food availability and shifted to alternative foods during low fruiting years caused by drought [45, 46].

We found that the frequency of high or moderate severity conflicts was greater within the Adirondack and Catskill Parks. This was likely a consequence of abundant bear habitat interspersed with low-density human development, which creates ample opportunity for bear access to human spaces and unsecured human-derived foods within these two parks (J. Hurst, personal observation). Most conflicts with black bears involve individuals investigating or habituating to human foods and trash in areas of high human use [13, 40, 47]. These conflicts may cause bolder behavior toward humans, which could result in increased conflicts with people [48–50]. The Adirondack and Catskill Parks encompass vast forest lands interspersed with villages and private lands, and there are many permanent and seasonal dwellings in each. This juxtaposition of wildland habitat and residential attractants can create heightened opportunity for human-bear conflicts and may differ from other large parks which have a hard wildland boundary and conflicts primarily in border communities.

We found an association between mild severity conflicts early in the year and high or moderate severity conflicts later in the year. Black bears are omnivorous [51], meaning that food-related conflicts with humans can be diverse, ranging from garbage and compost, to fruit trees and livestock [52]. Further reducing black bear access to anthropogenic foods and other attractants, non-lethal measures including electric fencing, modifying placement or configuration of field crops, and bear-resistant waste management should be used consistently and comprehensively in communities to reduce conflicts and facilitate coexistence between humans and bears [14, 53]. Additionally, a community can be involved in a Bear Smart Program with bylaws that requires garbage to be in bear-resistant containers, which in turn may result in reduced frequency of conflicts [54, 55].

We acknowledge several limitations with our study. We used coarse-resolution data (i.e., WMU-level) for conflict reports which made it difficult to link conflict incidence to finer resolution covariate data, such as land cover or human population density. We recommend that future reporting of human-black bear conflict data include finer-resolution location data (e.g., [56]). We also acknowledge that there is inherit variability within conflict classification due to differences in judgement among NYSDEC biologists and nuances in timing, location, and social perceptions of human-bear conflicts. Furthermore, we recognize that using bear harvest data as an index to bear abundance can be problematic, however, this was the only statewide data available. Many wildlife management agencies in the USA manage bear population sizes through harvest by adjusting harvest regulations to in turn mitigate human-bear conflicts [14, 57, 58].

Coexistence of people and wildlife is possible [59] and is a dynamic process [60]. Human behaviors can help facilitate coexistence between humans and wildlife, which in turn can mitigate conflicts [61–65]. Our findings demonstrate that variations in environmental factors including drought, and conflicts within parks early in the year could be a warning of increased frequency of high or moderate severity conflicts later in the year. As unsecured human foods and edible garbage are primary causes of human-black bear conflicts [28–31, 66], it is important to secure attractants (i.e., removing food sources or establishing physical barriers [62, 65, 67]) to facilitate coexistence of humans and black bears, which can mitigate the frequency and severity of conflicts.

## Supporting information

S1 Table Human-American black bear conflict dataset. (CSV)
